# A census of general surgery consultants in England and Wales: implications for the current and future surgical workforce

**DOI:** 10.1308/rcsann.2023.0018

**Published:** 2023-07-25

**Authors:** A Dosis, N Husnoo, S Roney, C Hendry, C Bonner, M Kronberga, E Moran, V Ninh, A Jha, T Grey, AK Saha

**Affiliations:** ^1^ Yorkshire and the Humber Deanery, UK; ^2^ University of Sheffield & Sheffield Teaching Hospitals NHS Foundation Trust, UK; ^3^ Calderdale and Huddersfield NHS Foundation Trust, UK; ^4^ St. Joseph’s Hospice, UK; ^5^ South Tees Hospitals NHS Foundation Trust, UK

**Keywords:** Diversity, Equality, General surgery, Workforce planning

## Abstract

**Introduction:**

This study aimed to describe the composition of the current general surgical consultant body in England and Wales and quantify levels of inequality within it as well as describe future workforce challenges

**Methods:**

This is an observational study of all general surgical departments in England and Wales. Consultant general surgeons were identified and data regarding their gender, country of undergraduate medical education, subspecialty and private practice were recorded.

**Results:**

Of the 2,682 consultant general surgeons in England and Wales identified for this study, just 17% are women, with gender inequality most marked in university teaching hospitals and among certain subspecialties. Almost 40% of consultants did not obtain their primary undergraduate degree in the United Kingdom and there are considerably fewer surgeons who studied abroad in university teaching hospitals. Over 40% of current general surgical consultants have been qualified for more than three decades and there is no equivalent sized group of younger consultants.

**Conclusions:**

There remains considerable gender and racial inequality in the consultant general surgical workforce, with pockets of a lack of diversity within university or teaching hospital surgical departments and some subspecialties. The proportion of surgeons in their fourth decade of clinical practice represents the largest group of current practising consultants, which points towards an impending workforce crisis should senior clinicians seek to reduce activity or consider taking early retirement.

## Introduction

The majority of surgical care in the United Kingdom (UK) occurs under a named consultant surgeon. As a result, consultant-led teams are crucial in the delivery of surgical care. However, there are limited data about the composition of the surgical workforce at a time when demand for consultant activity outstrips the demand for healthcare services.^1,2^

These issues are common to all surgical specialties; within general surgery, there are few details about the current consultant composition despite recent warnings about an ageing workforce^2^ and the impact of changes in the way that care is delivered meaning that more consultants are needed. It is also impossible to ignore the historical and widespread gender disparity among general surgery consultants and the under-representation of surgeons from a minority background.^3–7^

Although there has been a sustained increase in the number of women entering undergraduate medical education^8^ and, consequently, entering surgical training, women make up only 18% of consultant general surgeons.^3^ Although this is an increase from 6% three decades ago, surgery is still a specialty that struggles to recruit women.^9,10^ Several recent reports have also described the impact of racism within the health service^11^ and the under-representation of surgeons from a minority background in leadership positions.^12^

This study is a “census” of the consultant general surgical workforce in England and Wales, and aims to assess gender representation and determine the undergraduate educational background of consultants. Further, it aims to illustrate the relative age and stage of career of consultant surgeons to allow meaningful predictions to be made about workforce planning.

## Methods

All hospitals in England and Wales with a general surgical service were identified from the National Health Service (NHS) website.^13^ Each hospital entry was interrogated to find a listing of current consultant general surgeons. A comprehensive internet search was performed to determine the primary subspecialist interest of each surgeon and whether they had an active private practice. The accuracy of the data was checked by using local knowledge of the general surgical workforce from hospitals within Yorkshire and the Humber and comparing with the information presented on NHS websites.

Each surgeon was then identified on the General Medical Council national medical register of doctors^14^ and information about date of qualification, gender and university of primary qualification recorded. Each entry on the database was checked by two independent authors and, where there was disagreement about primary subspecialty, was discussed among the senior authors. Hospitals were split into two groups – tertiary and district general hospitals. The data set was correct as of 30 December 2021.

### Statistical analysis

Descriptive statistics were performed to test for significant associations between subspecialty and type of hospital with gender balance and university or country or primary origin. Consultants were stratified into groups according to length of time after primary medical undergraduate qualification, and further descriptive statistics were performed to test for significant associations between time after qualification and gender, subspecialty, private practice, and type of hospital. IBM SPSS Statistics, Version 26.0 (IBM Corporation, Armonk, NY, USA), was used for statistical analyses. Significance was defined as *p* < 0.05.

## Results

**Figure 1:**
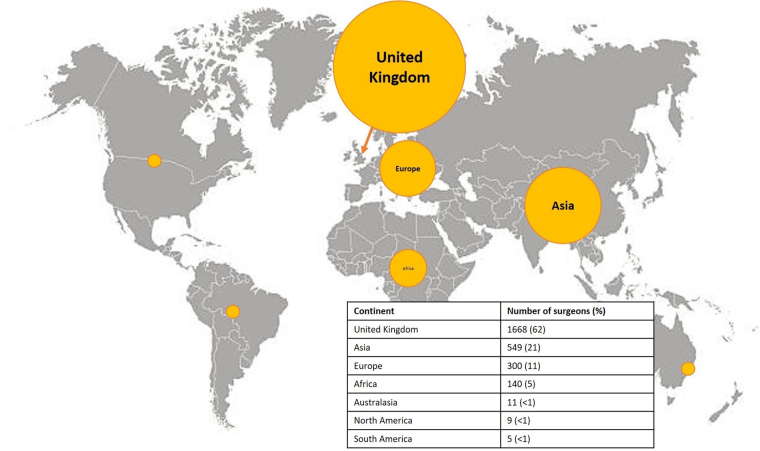
Continent of primary undergraduate medical qualification of consultant surgeons in England and Wales

There were 2,682 consultants, of whom 461 were women (17%). Most consultants (1,668, 62%) received their primary medical qualification from the UK, with Asia (549 consultants, 21%), Europe (300 consultants, 11%) and Africa (140 consultants, 5%) providing the majority of the other consultant surgeons (Figure 1). Sixty-two countries provided consultant surgeons in England and Wales; most received their primary undergraduate medical qualification from England (1,466 consultants, 55%), with the next most common countries of origin being India, Scotland and Pakistan (Table 1).

**Table 1:** Country of primary medical qualification for consultant surgeons in England and Wales

Country of primary qualification	Number of consultants	Per cent
England	1,466	54.7
India	364	13.6
Scotland	135	5.0
Pakistan	108	4.0
Egypt	64	2.4
Wales	61	2.3
Italy	59	2.2
Greece	48	1.8
Ireland	40	1.5
Nigeria	34	1.3
Iraq	27	1.0
Romania	25	0.9
Germany	19	0.7
South Africa	19	0.7
Poland	19	0.7
Hungary	18	0.7
Spain	16	0.6
Syria	15	0.6
Netherlands	14	0.5
Libya	13	0.5
Sri Lanka	12	0.4
Australia	9	0.3
Jordan	8	0.3
Malta	8	0.3
Jamaica	7	0.3
Czech Republic	7	0.3
Northern Ireland	6	0.2
Lithuania	4	0.1
Belgium	4	0.1
Ghana	3	0.1
Bangladesh	3	0.1
Bulgaria	3	0.1
Sudan	3	0.1
Portugal	3	0.1
Serbia	2	0.1
France	2	0.1
Myanmar	2	0.1
Argentina	2	0.1
Nepal	2	0.1
Brazil	2	0.1
Iran	2	0.1
Zimbabwe	2	0.1
Slovakia	2	0.1
New Zealand	2	0.1
Lebanon	1	0.0
Malaysia	1	0.0
Slovenia	1	0.0
Trinidad and Tobago	1	0.0
Mexico	1	0.0
Bosnia	1	0.0
Colombia	1	0.0
Tunisia	1	0.0
Ukraine	1	0.0
Turkey	1	0.0
Yemen	1	0.0
Palestine	1	0.0
Russia	1	0.0
Switzerland	1	0.0
Austria	1	0.0
Sweden	1	0.0
USA	1	0.0
China	1	0.0

There was widespread gender disparity (Table 2). This was most marked in tertiary hospitals when compared with district general hospitals; 13% of consultants in tertiary hospitals were women compared with 20% of consultants in district hospitals (*p* < 0.001); these differences persisted even among the youngest groups of surgeons (Table 3). Although the proportion of consultants who are women within each geographical region varied from 11% to 20%, there were no statistically significant differences in gender disparity between different regions.

**Table 2:** Consultant surgeons in England and Wales, stratified by gender, current hospital, country of undergraduate medical education, subspecialty, region and private practice

		Gender			Country of primary undergraduate qualification			Private practice		
Variable	Total *n* (%)	Male *n* (%)	Female *n* (%)	*p*-value	UK *n* (%)	Non-UK *n* (%)	*p*-value	Yes *n* (%)	No *n* (%)	*p*-value
Total	2,682	2,221 (83)	461 (17)		1,668 (62)	1,014 (38)		1,624 (61)	1,058 (39)	
Type of hospital										
Tertiary	1,049 (39)	912 (87)	137 (13)	0.001	688 (66)	358 (34)	0.003	642 (61)	404 (39)	0.521
District general	1633 (61%)	1309 (80%)	324 (20%)		980 (60)	653 (40)		982 (60)	651 (40)	
Subspecialty										
Colorectal	928 (34)	785 (85)	143 (15)	<0.001	631 (68)	297 (32)	<0.001	576 (62)	352 (38)	<0.001
Upper GI	458 (17)	422 (92)	36 (8)		288 (63)	170 (37)		318 (69)	140 (31)	
Breast	408 (15)	241 (59)	167 (41)		230 (56)	178 (64)		249 (61)	159 (39)	
HPB	96 (4)	90 (94)	6 (6)		53 (55)	43 (45)		67 (70)	29 (30)	
Vascular	473 (18)	422 (89)	51 (11)		310 (66)	163 (34)		289 (61)	184 (39)	
Endocrine	47 (2)	37 (79)	10 (2)		31 (66)	16 (34)		32 (68)	15 (32)	
Transplant	57 (2)	51 (90)	6 (10)		20 (35)	37 (65)		17 (30)	40 (70)	
General/Emergency	215 (8)	173 (80)	42 (20)		105 (49)	110 (51)		76 (35)	139 (65)	
Region										
East of England	221 (8)	181 (81)	40 (19)	0.449	125 (57)	96 (63)	<0.001	137 (62)	84 (38)	<0.001
Yorkshire/ Humber	298 (11)	250 (84)	48 (16)		183 (61)	115 (39)		161 (54)	137 (46)	
London	437 (16)	366 (84)	71 (16)		238 (54)	199 (46)		321 (73)	116 (27)	
Wessex	104 (4)	83 (80)	21 (20)		80 (77)	24 (23)		72 (69)	32 (31)	
West Midlands	304 (11)	239 (79)	55 (21)		214 (70)	90 (30)		173 (57)	131 (43)	
North West/Mersey	353 (13)	291 (82)	62 (18)		194 (55)	159 (45)		167 (47)	186 (53)	
South West	266 (10)	211 (79)	55 (21)		214 (80)	52 (20)		155 (58)	111 (42)	
Kent/Surrey/Sussex	202 (7)	168 (83)	34 (17)		126 (62)	76 (38)		145 (72)	57 (28)	
North East	167 (6)	149 (89)	18 (11)		108 (65)	59 (35)		73 (44)	94 (56)	
East Midlands	120 (4)	103 (86)	17 (14)		53 (44)	67 (56)		77 (64)	43 (36)	
Thames Valley	210 (7)	170 (81)	40 (19)		133 (63)	77 (37)		143 (68)	67 (32)	
Wales	87 (3)	75 (86)	12 (14)		57 (66)	30 (34)		59 (68)	28 (32)	
Gender										
Male	2,221 (83)	–	–	–	1,310 (59)	911 (41)	<0.001	1,465 (66)	756 (34)	<0.001
Female	461 (17)	–	–		358 (78)	103 (22)		159 (34)	302 (66)	
Country of undergraduate education										
UK	1,668 (62)	1,310 (79)	358 (21)	<0.001	–	–	–	993 (59)	675 (41)	0.166
Non-UK	1,014 (38)	911 (90)	103 (10)		–	–		631 (62)	383 (38)	
Private practice?										
Yes	1,624 (61)	1,465 (90)	159 (10)	<0.001	993 (61)	631 (39)	0.166	–	–	–
No	1,058 (39)	756 (71)	302 (29)		675 (64)	383 (36)		–	–	

GI = gastrointestinal; HPB = hepatopancreatobiliary

**Table 3:** Consultant surgeons in England and Wales, stratified by years since obtaining primary undergraduate medical qualification

	Gender/Type of hospital, *n* (%)						Subspecialty, *n* (%)							
Years after primary undergraduate qualification (*n*; %)	Male (*N* = 2,221)	Female (*N* = 461)	Tertiary hospital (*N* = 1,049)	District general hospital (*N* =1,633)	Female consultants in tertiary hospitals (*N* = 137)	Female consultants in district general hospital (*N* =324)	Colorectal (*N* =928)	Upper GI (*N* =458)	Breast (*N* =408)	HPB (*N* =96)	Vascular (*N* =473)	Endocrine (*N* =47)	Transplant (*N* =57)	General/Emergency (*N* =215)
10–14 (43; 2)	33	10	20	23	4 (20)	6 (27)	11 (1)	9 (2)	6 (1)	2 (2)	5 (1)	1 (2)	2 (4)	7 (3)
15–19 (384; 14)	279	105	151	233	26 (17)	79 (34)	125 (13)	66 (14)	62 (16)	11 (12)	48 (10)	7 (15)	7 (12)	52 (24)
20–24 (545; 20)	399	146	223	321	47 (21)	99 (30)	219 (24)	103 (22)	81 (20)	23 (24)	101 (21)	7 (15)	10 (18)	67 (31)
25–29 (555; 21)	468	87	228	327	29 (13)	58 (18)	227 (24)	95 (22)	82 (20)	25 (26)	91 (19)	4 (9)	12 (21)	19 (10)
30–34 (594; 22)	520	74	215	379	15 (7)	59 (16)	211 (22)	110 (24)	93 (23)	22 (23)	107 (23)	11 (23)	13 (23)	20 (9)
35–39 (341; 13)	315	26	144	197	10 (7)	16 (8)	93 (10)	50 (11)	40 (10)	8 (8)	79 (17)	11 (23)	6 (11)	35 (16)
>40 (220, 8%)	207	13	68	153	6 (4%)	7 (2%)	52 (6)	25 (5)	44 (10)	5 (5)	42 (9)	6 (13)	7 (11)	15 (7)
<25 years after primary medical qualification	711	261	394	577	77	184	355 (38)	178 (38)	149 (37)	36 (38)	154 (32)	15 (32)	19 (36)	126 (58)
>30 years after primary medical qualification	1,042	113	427	729	31	82	356 (38)	185 (40)	177 (43)	35 (36)	228 (49)	28 (59)	26 (45)	70 (32)

GI = gastrointestinal; HPB = hepatopancreatobiliary

There were marked gender disparities between subspecialties; 41% of consultant breast surgeons were women, but just 6% of hepato-pancreato-biliary (HPB) and 8% of upper gastrointestinal (GI) surgeons were women. These differences were more marked in tertiary hospitals, and several units did not have any women at all. There were significantly fewer female consultants in tertiary units compared with district hospitals in colorectal surgery (12% vs 40%, *p* = 0.041), HPB/upper GI surgery (6% vs 10%, *p* = 0.01) and breast surgery (31% vs 45%, *p* = 0.012).

There were widespread differences in the proportion of consultant surgeons who received their primary undergraduate medical qualification from a non-UK university (Table 2). Whereas over 40% of consultant surgeons in district hospitals were from a non-UK background, 34% of consultants in tertiary hospitals studied in non-UK universities (*p* = 0.003). There were also marked differences in subspecialty and region; whereas 32% of colorectal surgeons trained in non-UK universities, more than half the transplant and emergency/general surgeons were from non-UK universities (*p* < 0.001). Over half the consultant surgeons in the East Midlands were from non-UK universities, whereas just one-fifth of consultants in Wessex and the South-West studied outside the UK (*p* < 0.001).

The distribution of surgeons from date of primary medical qualification to censoring date was not uniform across the consultant workforce. There were 43 consultant surgeons (2%) who qualified from medical school between 10 and 15 years ago with 1,155 (43%) consultants having qualified more than 30 years ago (Table 3). The proportion of female surgeons was much greater among younger groups (Table 3); whereas 6% of surgeons who had qualified from medical school more than 40 years ago were female, 27% of surgeons who qualified less than 20 years ago were women (*p* < 0.001).

**Figure 2:**
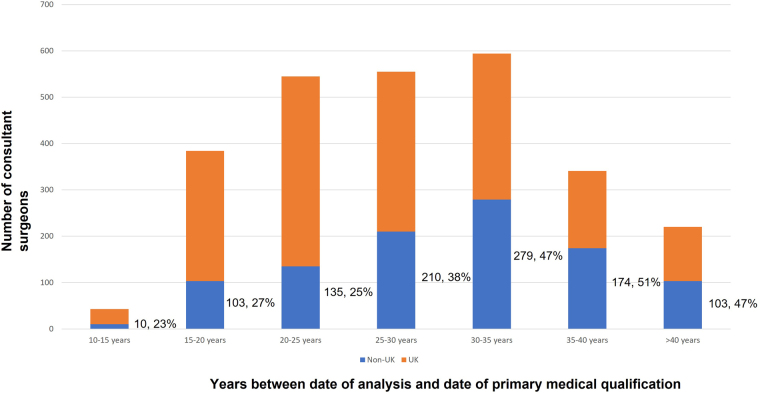
Proportion of consultant surgeons who received their primary medical qualification outside the UK, stratified by time from primary qualification

The proportion of surgeons from a non-UK background was greater among older surgical groups (Figure 2): 47% of older surgeons from a non-UK university compared with 24% of surgeons who qualified less than 20 years ago (*p* < 0.001). There were also significant differences in age of the consultant workforce and subspecialty (Table 3); whereas there was broad numerical equivalence between consultants in the first and last 15 years of practice in colorectal, upper GI and HPB surgery, there was a much greater proportion of older surgeons in vascular, breast and transplant surgery.

**Figure 3:**
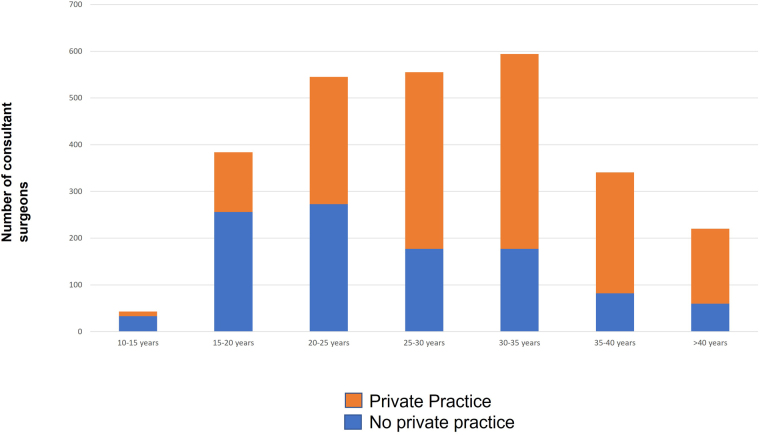
Proportion of consultant surgeons who have an active private practice, stratified by time from primary qualification

There were 1,624 (61%) consultant surgeons who had a private practice and this varied widely across regions and specialties (Table 2). Private practice was more common among men than women (66% vs 34%, *p* < 0.001) and varied across specialties; only 30% of transplant surgeons and 35% of general or emergency surgeons had a private practice compared with 70% of upper GI and HPB surgeons (*p* < 0.001). Although 73% of surgeons in London had a private practice, only 44% of surgeons in the North East (*p* < 0.001) had one. The proportion of surgeons with private practice was greater among older surgical groups (Figure 3).

## Discussion

This study describes stark trends in the composition of the consultant general surgical body in England and Wales, with implications for workforce planning. Over 40% of consultant surgeons have been qualified for more than 30 years, which has profound consequences if a proportion of this group seek early retirement or reduce activity. The service is sustained by consultants who studied outside the UK, with almost 40% of surgeons having gained their primary medical qualification from abroad; however, this finding must be juxtaposed against the falling number of surgeons who obtained their primary undergraduate qualification outside the UK, with fewer than a quarter of our youngest surgeons having studied abroad. There remain considerable gender disparities among consultant surgeons; of almost 3,000 consultant surgeons, just 17% were women. There is considerable variation throughout the country, with notable differences existing between tertiary (or university) hospitals and district general hospitals and between subspecialties.

Despite numerous recent studies,^3,4,15^ gender disparity in the consultant surgical workforce remains. Although there have been improvements, with female consultants increasing from 6% to 27%, there remain several areas of gender inequality. The most marked of these is between tertiary university hospitals and district general hospitals, with fewer female surgeons in university hospitals. Much has been made of the importance of gender balance in conferences, specialty associations and leadership positions,^12,16,17^ but these ignore the findings of this study which suggest the underlying problem is under-representation of women in tertiary hospitals. It is unusual for surgeons from district general hospitals to be in senior leadership positions, and it is unsurprising that there is such poor gender equality across the specialty when the standard bearers of our university hospitals are so unequal. Surgery remains a relatively unpopular career choice for medical students^18^ and the lack of gender equality and role models in university hospitals, which medical students have greatest exposure to, is unlikely to inspire them into a surgical profession.^19–21^ There were also considerable differences among subspecialties, and these were also most marked in tertiary hospitals; fewer than 10% of upper GI or HPB surgeons were female, and the most under-represented group were female surgeons who studied in non-UK universities.

Although these data show the current picture of consultant surgeons, there are some grounds for optimism in future cohorts of surgeons. Almost 40% of current higher surgical trainees are women, and it is anticipated that gender parity among registrars may be reached by 2028.^22^ However, this should be balanced against the fact that although the absolute number of female surgeons within younger cohorts is increasing, the proportion of women within university hospitals and certain subspecialties remains static. It remains to be seen whether the move towards gender parity among trainees is reflected across the entire consultant surgical workforce or whether current inequalities become entrenched if negative perceptions and experiences persist.^23,24^

The proportion of consultant surgeons in the UK who obtained their primary medical qualification outside the UK was over 40%, and this demonstrates the crucial role that doctors who studied abroad play in our NHS; indeed, previous workforce studies have described how the UK has not trained enough doctors to meet demand, with medical student places having fallen steadily since 2010 until recently.^25,26^ Whereas in previous decades, this shortfall was managed by “importing doctors”, as reflected in the higher proportion of older surgeons in this study who obtained their primary undergraduate qualification abroad, this has become harder as the global supply of highly trained doctors has fallen steadily; in 2019, the World Health Organization described a global shortage of two million doctors.^27^ These pressures may have been exacerbated by changes in governmental policy towards immigration; from 2008 to 2018, non-European Economic Area doctors required a tier 2 visa to practise in the UK, and these visas were limited to 20,700 per annum for all professions with doctors included in this cap.^26^

There were marked regional variations identified in this study which are difficult to ignore; these may be related to the population at large in those areas – the South-West of England, which had the least diverse consultant body in relation to country of primary qualification, is also among the most heavily White areas of the UK,^28^ in contrast to the East Midlands. In this study, there were considerable differences between university and district hospitals, with a much smaller proportion of surgeons in university hospitals having studied abroad. The impact of these differences is also highlighted in the composition of senior leadership positions in various surgical societies. Although over half of transplant and emergency general surgeons are from outside the UK, just one past president of the British Transplantation Society (BTS) and no past presidents from the Association of Surgeons of Great Britain and Ireland obtained their primary medical qualification from a non-UK university,^29,30^ highlighting a lack of representation of the consultant surgical workforce at senior levels, though the current executive committee and council of the BTS does demonstrate a much greater degree of diversity.

**Figure 4:**
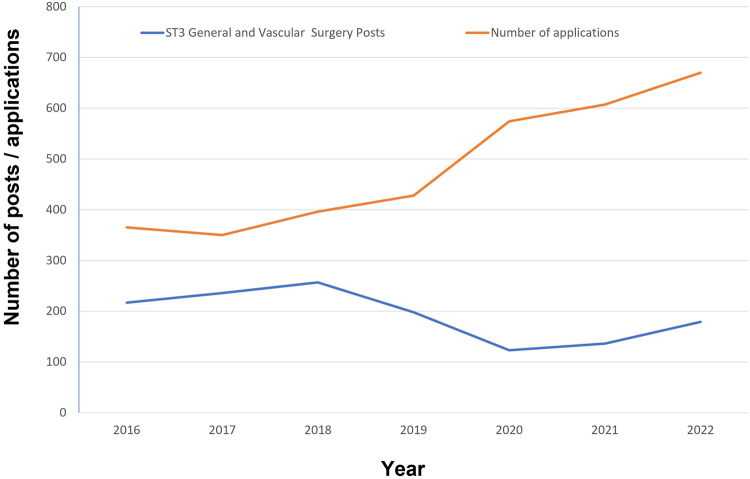
Number of applications and ST3 training posts for general and vascular surgery, 2016–2022

These data also question the viability of the consultant surgical workforce. Almost half the consultants in this study qualified from medical school more than 30 years ago and so could reasonably be thought to be in the final decade of practice; recent changes to pension taxation and working conditions have led to widespread reports of consultants seeking early retirement or reducing activity^31^ at a time when pressures on elective waiting lists^32^ and emergency surgical rotas is greater than ever; though some recent changes to pension taxation were introduced in the Spring Budget of 2023, overall policy has not addressed long-standing concerns regarding pay-restoration and working conditions. Adequate workforce planning would supply an equal, or greater, number of consultants to replace those who retire, but these data suggest that this is not currently the case. Despite the significant concerns about the loss of activity from the current consultant workforce, there have not been large scale increases in training numbers. Over the past six years, there has been a relative decline in ST3 general surgery training numbers^33^ (Figure 4); with broadly static, or falling, numbers of junior surgeons entering training, it is unlikely that even a small drop in current consultant numbers will be balanced by the number of new surgeons starting consultant practice.

These issues are not limited to surgery, with several “acute” specialties reporting unfilled consultant posts^31^ and unfilled rotas.^2^ Governmental policy to reduce the burden of pension taxation and improve pay and working conditions is critical to sustain safe services. These data also demonstrate that subspecialties are not affected equally; whereas there are broadly equal numbers of younger and older surgeons in colorectal, upper GI and HPB surgery, there are much greater proportions of older surgeons in breast, endocrine and transplant surgery, which may have implications for the viability of those subspecialties. The data on private practice also demonstrate marked differences; older, male surgeons from the south of England are more likely to have an active private practice, and surgeons in busier acute specialties, such as transplant or emergency surgery, are considerably less likely to conduct private practice.

### Study limitations

The methodology of this study has some limitations that limit the scope of the conclusions; the censoring date means that some new consultants are not recognised in this data set. Further, the method of identifying consultants, from each hospital’s NHS website, may miss some surgeons. However, these websites are the patient-facing portals for each hospital and so are as accurate a source of data as patients have available. In this study, the country of primary qualification has been used as a surrogate for ethnic background and this clearly has some flaws. This methodology ignores the background of British surgeons who studied abroad and surgeons from a minority background who studied in the UK, and conclusions on race and ethnicity should be drawn with caution. However, the implications from this study do reflect wider pieces of work and the lived experiences of current practising surgeons^11^ and cannot be ignored. There are no widely available ethnic background data at a specialty-specific level, and this methodology does allow for some discussion about the background of consultants in the UK.

Although this study accurately describes the current consultant general surgical workforce in England and Wales, it does not necessarily explain why such gender, racial and age disparities exist, though several studies have explored these areas.^9,10,34,35^ Commonly cited reasons for gender inequality, such as concerns about raising a family and unsocial working conditions, are not necessarily true; though female trainees may question the compatibility of raising a family and a surgical career, only one-third of women reported this as their main concern and only 10% described unsocial working hours as a significant barrier.^34^ Much more likely is the discrimination that women face in the surgical workplace and the blunt truth described in this study – women and surgeons from minority backgrounds are much less likely to hold consultant positions in university hospitals and these are the institutions that our medical students experience first, and our senior leadership positions are made up from.

There are many initiatives to improve the inequalities highlighted in this study; perhaps the most important and current of these is the Kennedy Report,^11^ commissioned and published by the Royal College of Surgeons of England (RCS England). The report described uncomfortable testimonies of sexism, racism and homophobia within surgery, but also demonstrated a genuine willingness to improve the diversity within the profession from our most senior leaders and a commitment to tangible and measurable aims with clear timescales to achieve change. RCS England, through its workforce group, has also committed to conducting a census of the surgical workforce across the specialties, which will give greater insight and knowledge about the current composition of the consultant body in the UK. These initiatives are both necessary and welcome, and should accelerate the positive changes that have been seen among trainees.

However, it is also incumbent on all surgeons and clinical leaders to recognise and challenge inequality; though individual hospitals are not named in this study, there are several university departments with very few or no female consultants. There is also clear under-representation of surgeons from a minority background, which in turn leads to inequality in our specialty associations; such inequality may also be present throughout healthcare and further work is necessary to explore the composition of the workforce for other specialties. Trainees and training programme directors should question why units and departments in their regions are as they are, and make attempts to ensure that all future colleagues are welcome and feel that they can progress in specialist surgical careers. Only by encouraging and supporting our current consultants and trainees will the profession be able to deal with the workforce challenges highlighted in this study and address the issues described herein; these data suggest that the timeframes involved may be short and the problems may be acute and as such, urgent solutions and progress are crucial to maintain the future viability of the consultant general surgical workforce in the UK.

## Author contributors

A.D., N.H., T.G. and A.K.S. planned and designed the study, conducted the data collection team and prepared the manuscript. A.D., N.H., S.R., C.H., V.N., A.J., C.B., M.K. and E.M. collected the data and conducted the study. A.D., N.H., T.G. and A.K.S. checked the data and performed the statistical analyses. A.K.S. is overall guarantor of the content and accepts full responsibility for the work and the conduct of the study, had access to the data and controlled the decision to publish. The corresponding author attests that all listed authors meet authorship criteria and that no others meeting the criteria have been omitted.

## Data availability

Full raw data are available from the corresponding author (Arin Saha, arin.saha@cht.nhs.uk) upon any request.

## Transparency statement

The lead author affirms that this manuscript is an honest, accurate and transparent account of the study being reported; that no important aspects of the study have been omitted; and that any discrepancies from the study as planned (and, if relevant, registered) have been explained.
